# Antitumor Effects and Biological Mechanism of Action of the Aqueous Extract of the *Camptotheca acuminata* Fruit in Human Endometrial Carcinoma Cells 

**DOI:** 10.1155/2014/564810

**Published:** 2014-05-21

**Authors:** Chi-Shian Lin, Pin-Chien Chen, Chien-Kai Wang, Chia-Woei Wang, Yu-Jia Chang, Cheng-Jeng Tai, Chen-Jei Tai

**Affiliations:** ^1^Graduate Institute of Medical Sciences, College of Medicine, Taipei Medical University, Taipei 110, Taiwan; ^2^Department of Surgery, Chi Mei Hospital Chiali, Tainan 170, Taiwan; ^3^Department of Obstetrics and Gynecology, School of Medicine, College of Medicine, Taipei Medical University, Taipei 110, Taiwan; ^4^Division of Hematology and Oncology, Department of Internal Medicine, Taipei Medical University Hospital, 252 Wu Hsing Street, Taipei 110, Taiwan; ^5^Department of Internal Medicine, School of Medicine, College of Medicine, Taipei Medical University, Taipei 110, Taiwan; ^6^Department of Chinese Medicine, Taipei Medical University Hospital, 252 Wu Hsing Street, Taipei 110, Taiwan; ^7^Department of Obstetrics and Gynecology, Taipei Medical University Hospital, Taipei 110, Taiwan; ^8^Cancer Research Center, Taipei Medical University and Hospital, Taipei 110, Taiwan; ^9^Department of Surgery, Taipei Medical University and Hospital, Taipei 110, Taiwan; ^10^Division of General Surgery, Department of Surgery, Taipei Medical University Hospital, Taipei 110, Taiwan; ^11^Graduate Institute of Clinical Medicine, College of Medicine, Taipei Medical University, Taipei 110, Taiwan; ^12^Traditional Herbal Medicine Research Center, Taipei Medical University Hospital, Taipei 110, Taiwan

## Abstract

The aqueous extracts of the leaves and fruit of *Camptotheca acuminata* have long been used in traditional Chinese medicine (TCM) for treating cancer patients. The chemotherapeutic drug, camptothecin (CPT), and related analogs were first isolated from *C. acuminata* in the 1970s. Although the antitumor effects of CPT have been characterized in recent years, the antitumor effects of aqueous extracts of *C. acuminata* have not been clarified. The aims of our current study were to determine the tumor-suppression efficiency of an aqueous extract of the fruit of *C. acuminata* (AE-CA) in the human endometrial carcinoma cell lines, HEC-1A, HEC-1B, and KLE, and compare its antitumor effects with those of CPT. Cell viability assays indicated that a dosage of AE-CA containing 0.28 mg/mL of CPT demonstrated enhanced cytotoxicity, compared with CPT treatment. The effects of AE-CA on the induction of cell cycle arrest, the accumulation of cyclin-A2 and -B1, and the activation of caspase-3 and caspase-7 were similar to those of CPT. Furthermore, AE-CA exhibited a synergistic effect on the cytotoxicity of cisplatin in HEC-1A and HEC-1B cells. These results indicated that AE-CA is a potent antitumor agent and can be combined with cisplatin for the treatment of human endometrial cancer.

## 1. Introduction

Endometrial cancer is a highly prevalent gynecological malignancy worldwide [1, 2]. Radiotherapy and chemotherapy are the primary treatment approaches for advanced and recurrent endometrial cancer. The prognosis for such cases is poor, with a survival rate of approximately 20% [3, 4]. The common chemotherapeutic regimens, which include platinum analogs, doxorubicin, taxanes, and camptothecin (CPT) analogs, are recommended for treating endometrial cancer patients. However, the combination of suboptimal response rates, adverse effects, and the development of drug resistance during the treatment period highlight the urgent need for alternative approaches for improving the clinical outcomes in endometrial cancer patients.

In traditional Chinese medicine (TCM), a number of formulas are considered to be effective treatments for cancer, but the exact tumor-suppression efficacy of such formulas and their biological mechanisms is unclear. Therefore, appropriate preclinical evaluations are required to clarify the antitumor effects of these TCM formulas and facilitate the development of novel alternative chemotherapeutic approaches for treating endometrial cancer.* Camptotheca acuminata *flourishes in southern China and Taiwan and has long been used in TCM. The anticancer drug, CPT, was first isolated from* C. acuminata *in the 1960s, and an abundance of CPT is present in the leaves, fruit, seeds, and bark of the plant [5, 6]. The aqueous extract of the fruit of* C. acuminata* (AE-CA) is administrated orally to cancer patients by TCM practitioners. The main active component, CPT, is responsible for the antitumor effects of AE-CE.

The anticancer biological mechanism of CPT and CPT analogs involves the inhibition of topoisomerase I, which is required for DNA replication in mammal cells [7]. In tumor cells, CPT analogs arrest the DNA replication fork and cause double-stranded DNA breakage, which induces apoptosis [8, 9]. The induction of apoptosis by CPT likely occurs through the activation of caspase-3 and caspase-7 [10]. Two or three chemotherapeutic drugs are often administrated in combination to improve the clinical efficacy of chemotherapy regimens. Among such combined chemotherapeutic strategies, CPT analogs are often used with platinum-based analogs or taxanes [11–13]. One CPT analog, irinotecan, has been shown to improve clinical outcomes in advanced endometrial cancer patients when used in combination with carboplatin [14].

Although the antitumor effects of CPT analogs have been quantified in recent years, the tumor suppression efficacy of AE-CA has not been subjected to an evidence-based evaluation. The main aims of this study were to determine the antitumor efficacy of AE-CA for endometrial cancer and clarify whether the biological mechanism underlying the antitumor effects of AE-CA is consistent with those previously determined for CPT. Because a regimen of CPT combined with the platinum analog, cisplatin, has been shown to be effective for treating endometrial cancer in humans, the antitumor effects of AE-CA combined with cisplatin were assessed in the human endometrial-cancer cell lines, HEC-1A, HEC-1B, and KLE.

## 2. Materials and Methods

### 2.1. Preparation of AE-CA

The AE-CA was purchased from Sun-Ten Pharmaceutical (Taipei, Taiwan). In accordance with the information provided by the manufacturer, 50 g of the dried fruit of* C. acuminata* was immersed in 750 mL of distilled water. The mixture was gradually heated to 100°C over a period of 50 min and boiled until the volume of the liquid was reduced to 50 mL to obtain an extract with a final concentration of 1 g/mL, based on the weight of the initial dried material. According to the datasheet provided by manufacturer, the AE-CA contained 0.28 mg/mL of CPT.

### 2.2. Cell Culture and Cytotoxicity Analysis

The human endometrial cell lines, KLE, HEC-1A, and HEC-1B, were used for our evaluation of the AE-CA, all of which were purchased from American Type Culture Collection (ATCC, Manassas, VA, USA). The HEC-1A and HEC-1B cell lines are moderately differentiated, estrogen-dependent adenocarcinoma cells. By contrast, the KLE cells are poorly differentiated, estrogen-independent adenocarcinoma cells. The human lung fibroblast cell line, WI-38 (Food Industry Research Development Institute, Hsinchu, Taiwan), was used as a control to demonstrate the effects of the AE-CA on noncancerous human cells. All of the cells were maintained in Dulbecco's modified Eagle's medium/nutrient mixture F-12 (Invitrogen, Carlsbad, CA, USA) containing 100 U/mL of penicillin (Invitrogen) and 100 *μ*g/mL of streptomycin (Invitrogen) at 37°C in 5% CO_2_ in a humidified incubator.

To evaluate the cytotoxicity of the AE-CA, the HEC-1A and HEC-1B cells were seeded in 96-well microplates at a density of 5 × 10^3^ cells per well. The KLE cells were seeded at a density of 1 × 10^4^ per well. After an overnight incubation, the HEC-1A, HEC-1B, and KLE cells were treated with 0.01 to 5 mg/mL of AE-CA or 0.05 to 1 *μ*M CPT for 24 or 48 h. To evaluate the combination effect of AE-CA with cisplatin, HEC-1A cells were treated using 0 to 50 *μ*M cisplatin and 0.25 mg/mL of AE-CA or 0.2 *μ*M CPT. HEC-1B were treated using 0 to 50 *μ*M cisplatin and 0.625 mg/mL of AE-CA or 0.5 *μ*M CPT. Cell viability was determined using a 3-(4, 5-dimethylthiazol-2-yl)-2, 5-diphenyltetrazolium bromide (MTT) assay. A 30 *μ*L aliquot of 5 mg/mL of MTT (Bio Basic, Markham, Ontario, Canada) in phosphate-buffered saline (PBS) was added to each well, and the cells were incubated at 37°C for an additional 3 h. The contents of each well were replaced with 100 *μ*L of dimethyl sulfoxide (JT Baker, Phillipsburg, NJ, USA), and the plates were shaken at room temperature for 15 min in the dark to dissolve the formazan product. The optical density of each well was measured at 550 nm, using an EMax^*Ⓡ*^ microplate reader (Molecular Devices, Sunnyvale, CA, USA). The concentration at which 50% of the cells remained viable (IC_50_) was calculated using the CalcuSyn software (BioSoft, Cambridge, UK).

### 2.3. Cell Cycle Analysis

HEC-1A and HEC-1B cells were seeded in 6 cm culture dishes at a density of 5 × 10^5^ cells per dish, and the KLE cells were seeded at a density of 1 × 10^6^ per dish. After a 24 h incubation, the cells were treated with 0.2 mg/mL of AE-CA, 0.16 *μ*M CPT, or an equivalent volume of the control medium for 48 h. The cells were harvested by adding 0.05% trypsin-EDTA (Life Technologies, Carlsbad, CA, USA), and the plates were centrifuged to remove the supernatant. The cell pellets were gently washed with 1 mL of PBS and fixed in 4 mL of 75% ethanol before storage at −20°C overnight. The fixed cells were centrifuged to remove the ethanol and washed with PBS at room temperature. The cell pellets were suspended in 5 mL of PBS. The cells were stained by adding 1 mL of propidium iodide buffer containing 0.2 mg/mL of RNase A, 0.1% Triton X-100, and 20 *μ*g/mL of propodium iodide (Invitrogen, Carlsbad, CA, USA), and the cell suspension was incubated at room temperature for 15 min. The stained cells were examined using a FACScan flow cytometer (BD Biosciences, San Jose, CA, USA), and the flow cytometry data were analyzed using the CellQuest software (BD Biosciences, San Jose, CA, USA).

### 2.4. Immunoblotting Assay

The HEC-1A and HEC-1B cells were seeded in 6 cm culture dishes at a density of 5 × 10^5^ cells per dish, and the KLE cells were seeded at a density of 1 × 10^6^ per dish. After an overnight incubation, the HEC-1A and HEC-1B cells were treated with 0.2 mg/mL of AE-CA or 0.16 *μ*M CPT for 48 h, and the KLE cells were treated with 2 mg/mL of AE-CA or 1.6 *μ*M CPT for 48 h. Total protein lysates were prepared by suspending the cells in RIPA buffer containing 150 mM NaCl, 50 mM Tris-HCL (pH 7.5), 1% Nonidet P-40, 0.5% deoxycholate, 0.1% sodium dodecyl sulfate (SDS), 1 mM phenylmethanesulfonyl fluoride, 10 *μ*g/mL of leupeptin, and 100 *μ*g/mL of aprotinin. The concentration of total protein in the cell lysates was measured using a Bio-Rad Protein Assay Kit (Hercules, CA, USA). Aliquots of each cell lysate containing 30 *μ*g of total protein were subjected to SDS-polyacrylamide gel electrophoresis on a 12% acrylamide gel and transferred to a polyvinylidene fluoride membrane (Pall Corp, Port Washington, NY, USA). The membranes were probed using a primary antibody generated against one of the following proteins at the dilution indicated for each: cyclin-A2 (1 : 2000), cyclin-B1 (1 : 2000), caspase-3 (1 : 1000), caspase-7 (1 : 1000), and glyceraldehyde 3-phosphate dehydrogenase (GAPDH, 1 : 5000). All of the primary antibodies were purchased from Cell Signaling Technology (Danvers, MA, USA), except for the anti-GAPDH antibody, which was purchased from Abfroniter (Seoul, South Korea). Primary antibody reactivity was detected using a horseradish-peroxidase-conjugated donkey anti-rabbit secondary antibody (Santa Cruz Biotechnology, Dallas TX, USA) and visualized using the WesternBright ECL Western Blotting Detection Kit (Advabsta, Menlo Park, CA, USA). The intensity of the immunoreactive protein bands was measured using the ImageJ software (National Institutes of Health, Bethesda, MD, USA).

### 2.5. Statistical Analysis

The Student's* t*-test was performed to examine the statistical significance of the differences between the levels of caspase-3 and caspase-7 activation. The statistical analysis was performed using the SPSS software (IBM, Armonk, NY, USA). The results of comparisons with *P* < 0.05 were considered to represent statistically significant differences. The effects of AE-CA or CPT combined with cisplatin were evaluated using the CalcuSyn software, and a combination drug index (CDI) was constructed based on the Chou-Talalay median-effect method [15] to identify additive, antagonistic, or synergistic effects [16–18].

## 3. Results

### 3.1. AE-CA Inhibits the Growth of Human Endometrial Carcinoma Cells

**Figure 1:**
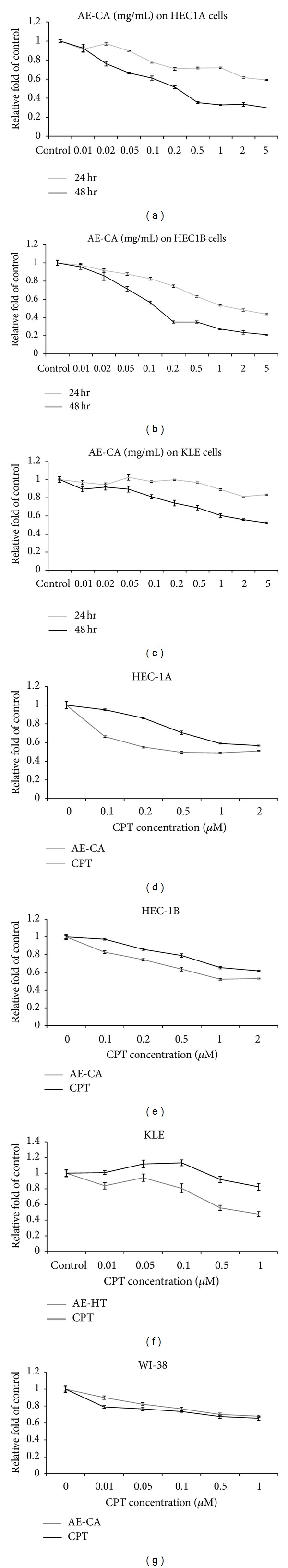
Cytotoxicity of AE-CA on the human endometrial carcinoma cell lines, HEC-1A, HEC-1B, and KLE, and the noncancerous human cell line, WI-38. (a–c) HEC-1A, HEC-1B, and KLE cells were treated with 0 to 5 mg/mL of AE-CA for 24 or 48 h. (d–g) HEC-1A, HEC-1B, KLE, and WI-38 cells were treated using the indicated doses of AE-CA and an equivalent concentration of CPT (*μ*M), based on 0.28 mg of CPT per milliliter of AE-CA. Cell viability was assessed using an MTT assay. The data are presented as the mean ± standard deviation.

To evaluate the antiproliferative effect of AE-CA on human endometrial carcinoma cells, the HEC-1A, HEC-1B, and KLE cell lines were treated with various amounts of AE-CA for 24 and 48 h. As indicated in Figures 1(a) to 1(c), the AE-CA treatment decreased the viability of the HEC-1A, HEC-1B, and KLE cells, and the reduction in viability was both dose- and time-dependent. The IC_50_ values for the HEC-1A, HEC-1B, and KLE cells treated with AE-CA for 24 h were 0.268, 0.339, and 1.611 mg/mL, respectively (Table 1). The antiproliferative effect of AE-CA varied according to cell type, with the HEC-1A and HEC-1B cells demonstrating greater sensitivity to AE-CA, compared with the KLE cells.

**Figure 2:**
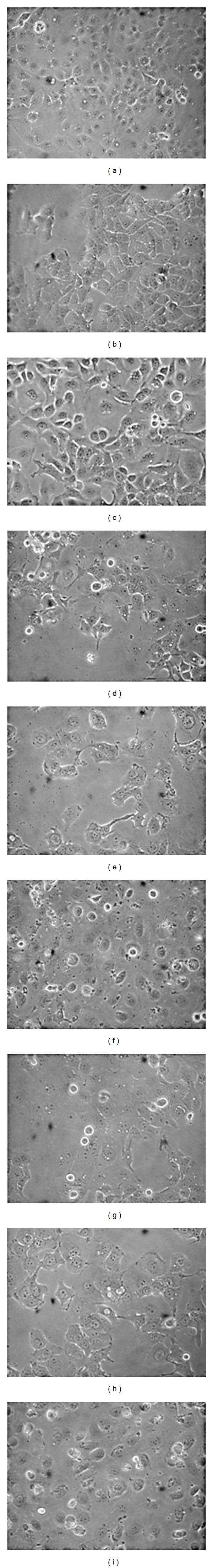
Morphological changes in human endometrial carcinoma cells induced by treatment using AE-CA or an equivalent concentration of CPT. HEC-1A, HEC-1B, and KLE cells were treated using a control culture medium (a–c); 0.2 mg/mL of AE-CA (d–f); or 0.16 *μ*M CPT (g–i) for 48 h (100x magnification).

We also compared the antiproliferative effect of a 48 h treatment using AE-CA to that of CPT, and the human lung fibroblast cell line, WI-38, was also tested to compare the cytotoxic effect of AE-CA on noncancerous human cells with that of CPT. The results indicated that AE-CA exerted a greater antiproliferative effect on all of the cell lines, compared with an equivalent amount of CPT (Figures 1(d) to 1(g)). No difference in cell morphology was observed between the AE-CA- and CPT-treated cells (Figure 2).

### 3.2. Cell Cycle Arrest and Apoptosis Are Induced by AE-CA

**Figure 3:**
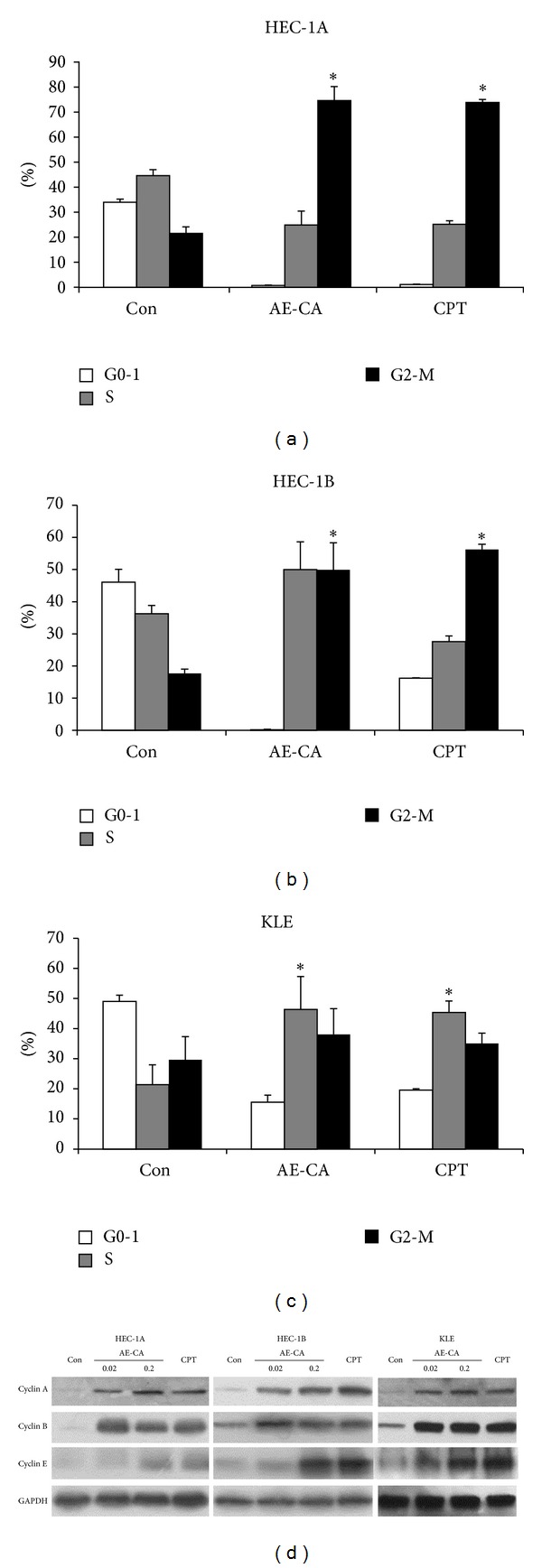
Effects of treatments using AE-CA or an equivalent concentration of CPT on cell-cycle arrest and the expression of cell-cycle protein markers in human endometrial carcinoma cells. (a) HEC-1A, (b) HEC-1B, and (c) KLE cells were treated using control medium, 0.2 mg/mL of AE-CA, or 0.16 *μ*M CPT. The data are presented as the mean ± standard deviation (*Student's *t*-test, *P* < 0.05). (d) HEC-1A, HEC-1B, and KLE cells were treated using AE-CA (0.02 or 0.2 mg/mL) or CPT (0.16 *μ*M) for 48 h, and the expression of cyclin-A2 and -B1 was examined using western blotting.

In cells, CPT acts as a topoisomerase-I poison, inducing S phase delay and G2/M arrest in many types of tumor cell [19]. Therefore, we performed a cell cycle analysis of HEC-1A, HEC-1B, and KLE cells treated with AE-CA or an equivalent amount of CPT for 48 h. The HEC-1A and HEC-1B cells treated using 0.2 mg/mL of AE-CA or 0.16 *μ*M CPT exhibited a higher percentage of cells in the G2/M phase, compared with the control cells (Figures 3(a) and 3(b)). By contrast, both AE-CA and CPT increased the percentage of KLE cells in the S phase (Figure 3(c)).

Although the cell-cycle-stage distributions for the HEC-1A and KLE cells treated with AE-CA were similar to those of the HEC-1A and KLE cells treated with CPT, the cell-cycle-stage distributions for the HEC-1B cells differed. The AE-CA treatment increased the percentage of HEC-1B cells in S phase and decreased the percentage of HEC-1B cells in the G0-1 phase. By contrast, the CPT treatment reduced the percentage of HEC-1B cells in both the S and G0-1 phases. The accumulation of cyclin-A2 and -B1 in the HEC-1A and HEC-1B cells treated with AE-CA or CPT also suggested that a G2/M cell cycle arrest had occurred (Figure 3(d)). In the KLE cells, although AE-CA and CPT also induced the accumulation of cyclin-A2 and -B1, neither treatment caused G2/M arrest (Figure 3(d)). In general, the dose- and time-dependent effects of AE-CA on cell-cycle regulation and the accumulation of cyclin-A2 and -B1 were similar to those of CPT.

**Figure 4:**
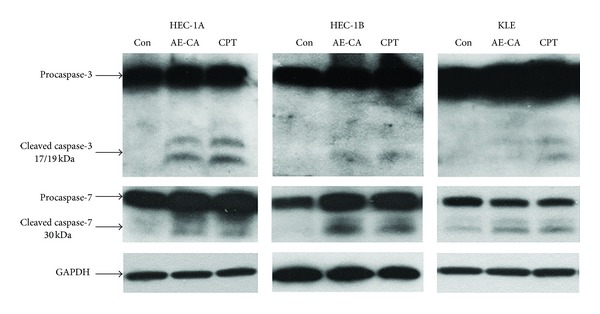
Activation of caspase-3 and caspase-7 in CPT- and AE-CA-treated human endometrial carcinoma cells. HEC-1A and HEC-1B cells were treated with 0.2 mg/mL of AE-CA or 0.16 *μ*M CPT for 48 h. KLE cells were treated with 2 mg/mL of AE-CA or 1.6 *μ*M CPT for 48 h. Procaspase-3 and procaspase-7 and their cleavage products were detected using western blotting.

The induction of apoptosis by CPT occurs through the activation of caspase-3 and caspase-7 [10]. Apoptotic-like morphological changes were observed in both the CPT- and AE-CA-treated endometrial carcinoma cell lines (Figure 2). Therefore, the activation of caspase-3 and caspase-7 was investigated in the HEC-1A, HEC-1B, and KLE cells treated with CPT or AE-CA (Figure 4). In the HEC-1A and HEC-1B cells, the activation of caspase-3 and caspase-7 in the CPT- and AE-CA-treated cells was similar. By contrast, a higher level of the 17- and 19-kDa fragments produced by caspase-3 cleavage accumulated in the KLE cells treated with CPT, whereas caspase-7 cleavage was similar between the CPT- and AE-CA-treated cells. These results suggested that both AE-CA and CPT activated caspase-3 and caspase-7 cleavage in the HEC-1A, HEC-1B, and KLE cells.

### 3.3. AE-CA-Enhanced Cisplatin Induced Cytotoxicity in Human Endometrial Carcinoma Cells

**Figure 5:**
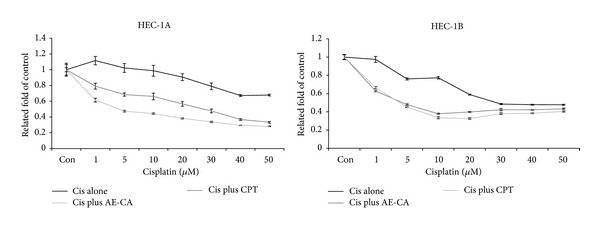
The cytotoxic effects of AE-CA or CPT used in combination with cisplatin. HEC-1A cells were treated using 0 to 50 *μ*M cisplatin and 0.25 mg/mL of AE-CA or 0.2 *μ*M CPT. HEC-1B were treated using 0 to 50 *μ*M cisplatin and 0.625 mg/mL of AE-CA or 0.5 *μ*M CPT. Cell viability was assessed using an MTT assay.

In combination chemotherapy for gynecological cancers, CPT analogs are often administered with platinum analogs, such as cisplatin. To examine the cytotoxic effects of AE-CA combined with cisplatin, HEC-1A and HEC-1B cells were treated with various concentrations of cisplatin alone or various concentrations of cisplatin combined with AE-CA or CPT for 48 h. The KLE cells were excluded from this evaluation because they had exhibited a low level of sensitivity to cisplatin in our preliminary analysis (data not shown). The AE-CA doses used to treat the HEC-1A and HEC-1B cells (0.25 and 0.625 mg/mL, resp.) were based on the concentrations of AE-CA that reduced HEC-1A and HEC-1B cell proliferation to approximately 40% of that of the control, and the CPT dose was based on the CPT content of the AE-CA dose. In both HEC-1A and HEC-1B cells, cisplatin combined with either AE-CA or CPT produced greater cytotoxic effects than those observed using cisplatin alone (Figures 5(a) and 5(b)). In the HEC-1A cells, the cytotoxicity of AE-CA combined with cisplatin was greater than that of CPT combined with cisplatin (Figure 5(a)). The treatments using cisplatin and AE-CA or cisplatin and CPT resulted in lower IC_50_ values (4.101 and 20.969 *μ*M, resp.) in the HEC-1A cells than that observed using cisplatin alone (60.114 *μ*M) (Table 1). No significant difference was observed between the enhanced cytotoxic effects of the combination treatments in the HEC-1B cells (Figure 5(b)). The CDI analysis indicated that both AE-CA and CPT demonstrated a synergistic effect in the HEC-1A and HEC-1B cells when combined with cisplatin (1–50 *μ*M) (Table 2). These results collectively indicated that the effects of AE-CA in human endometrial carcinoma cells were similar to those of CPT, whether used alone or in combination with cisplatin.

## 4. Discussion

In our current study, the antitumor effects of AE-CA were compared with those of CPT in the human endometrial carcinoma cell lines, HEC-1A, HEC-1B, and KLE. AE-CA tended to exhibit enhanced cytotoxicity in endometrial carcinoma cells, relative to equivalent concentrations of CPT, but its cytotoxic effects were similar to those of CPT in the noncancerous human cell line, WI-38 (Figures 1(d) to 1(g)). The enhanced cytotoxicity of AE-CA in endometrial carcinoma cells was likely caused by the presence of other CPT derivates in the AE-CA, such as hydroxycamptothecin and methoxycamptothecin, both of which have been identified in AE-CA preparations and shown to have greater antitumor activity than CPT [6, 20–22]. Because the cell-cycle analysis and investigation of caspase-3 and caspase-7 activation indicated no significant overall difference between the effects of AE-CA and CPT, the enhanced cytotoxicity of AE-CA might also be the result of unidentified components of the AE-CA that exert additive antitumor effects in human endometrial carcinoma cells.

Functioning as a topoisomerase-I poison, CPT disrupts DNA replication and leads to cell cycle arrest in the S, G1, or G2 phases [23, 24]. In the HEC-1A and HEC-1B cells, both the AE-CA and CPT treatments resulted in a significant increase in the percentage of cells in the G2/M phase. Furthermore, the AE-CA and CPT treatment also increased the percentage of cells in the S phase to approximately twice in KLE cells. The cell-cycle-stage distributions for the HEC-1A and KLE cells treated with AE-CA were similar to those of the HEC-1A and KLE cells treated with CPT, suggesting that the AE-CA-induced cell-cycle arrest was based on the biological activity of CPT. However, in the HEC-1B cells, AE-CA produced a greater increase in the percentage of HEC-1B cells in the S phase, compared with that observed for the CPT treatment, indicating that AE-CA might exert alternative effects on cell-cycle regulation in HEC-1B cells. The expression of cyclins A, B, and E is induced during cell-cycle arrest in CPT-treated cells [25, 26]. Our western blotting analysis demonstrated the accumulation of cyclin-A2 and -B1 in both the CPT- and AE-CA-treated cells, which indicated that the biological activity of AE-CA and CPT in human endometrial carcinoma cells is approximately equivalent.

CPT triggers apoptosis in tumor cells through the arrest of DNA replication forks and DNA breakage through the activation of caspase-3 and caspase-7 [10, 27]. Therefore, the cleavage of caspase-3 and caspase-7 provides protein markers for identifying apoptotic processes in endometrial carcinoma cells treated with CPT or AE-CA. In the AE-CA-treated HEC-1A, HEC-1B, and KLE cells, the activation of caspase-3 and caspase-7 was similar to that observed in the cells treated with an equivalent concentration of CPT. The cleavage of caspase-3 and caspase-7 was observed in both the CPT- and AE-CA-treated HEC-1A cells. By contrast, the cleavage of caspase-3 was barely detectable in the HEC-1B and KLE cells treated with CPT or AE-CA, whereas caspase-7 cleavage was clearly identified. These results indicated that cleavage of caspase-3 and caspase-7 is cell-type dependent and that the effects of AE-CA and CPT on the induction of apoptosis in human endometrial cells are similar. These results were consistent with our analysis of cell-cycle arrest and the expression of cyclin-A2 and -B1 in cells treated using AE-CA or CPT and suggested that the tumor-suppression efficiency of AE-CA in human endometrial carcinoma cells was equal to that of an equivalent concentration of CPT.

Because the antitumor effects of AE-CA were similar to those of an equivalent concentration of CPT, AE-CA might contain other components that can be used in combination with other chemotherapeutic drugs to treat human endometrial cancer. As a first-line chemotherapeutic drug, CPT can be administered alone or in combination with other antitumor drugs, such as taxanes or platinum analogs, for the treatment of human endometrial carcinomas [14]. Therefore, we also investigated the cytotoxic effects of AE-CA and cisplatin used in combination. The CDI analysis indicated that AE-CA and CPT exerted similar synergistic effects on the cytotoxicity of cisplatin, and the IC_50_ analysis also indicated that both AE-CA and CPT enhanced cytotoxicity of cisplatin in HEC-1A and HEC-1B cells. However, in HEC-1A cells, the cytotoxic effect of AE-CA and cisplatin combined was greater than that of CPT and cisplatin combined. These results collectively indicated that AE-CA contains a component that enhances the cytotoxicity of cisplatin in human endometrial cancer cells to a level above that observed for treatment using CPT and cisplatin in combination. Thus, AE-CA represents a potential alternative to CPT for combination chemotherapy using platinum analogs or other chemotherapeutic drugs.

## 5. Conclusion

**Figure 6:**
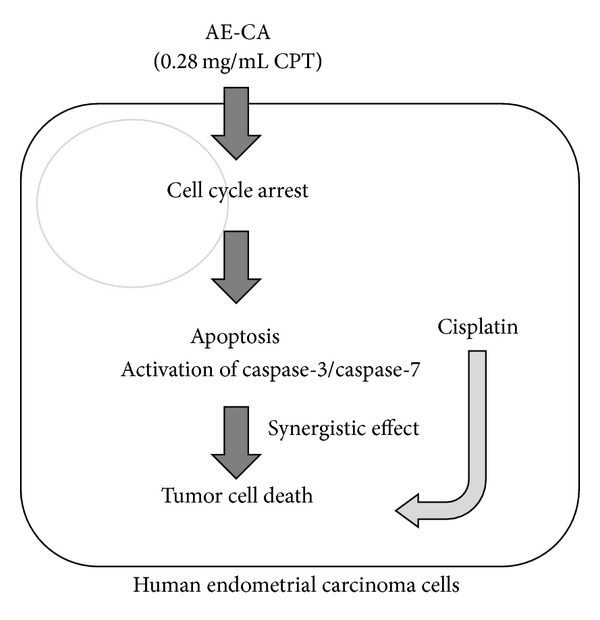
Summary of the antitumor effects of the AE-CA in human endometrial carcinoma cells.

In this study, we evaluated the antitumor effects of AE-CA in human endometrial carcinoma cells. The antitumor effects of AE-CA were similar to those of CPT, which is the most abundant antitumor component of AE-CA. The effects of AE-CA and CPT on the induction of apoptosis through cell-cycle arrest and the activation of caspase-3 and caspase-7 were also similar. Furthermore, AE-CA synergistically enhanced the cytotoxicity of cisplatin in human endometrial carcinoma cells (Figure 6). In conclusion, AE-CA represents a potential antitumor treatment for human endometrial carcinomas that can be used alone or in combination with platinum analogs, such as cisplatin.

## Figures and Tables

**Table 1:** Evaluation of tumor suppression efficiency of AE-CA on human endometrial carcinoma cells using the 50% inhibitory concentration (IC_50_).

	HEC-1A				HEC-1B			KLE
AE-CA (mg/mL)	0.268				0.339			1.611

In comparison of AE-CA and CPT								
	Con	Plus AE-CA	Plus CPT		Con	Plus AE-CA	Plus CPT	

Cisplatin (*μ*M)	60.114	4.101	20.969		33.111	4.879	4.013	

IC_50_ values for AE-CA in HEC-1AHEC-1A, HEC-1B, and KLE cells were calculated using the CalcuSyn software and based on the cytotoxicity data described in Figure 1.

IC_50_ values for cisplatin alone (Con), cisplatin with AE-CA (plus AE-CA), and cisplatin with CPT (plus CPT) were calculated using the CalcuSyn software and based on the cytotoxicity data described in Figure 5.

**Table 2:** The cytotoxic effects of cisplatin combined with AE-CA or CPT in HEC-1A and HEC-1B cells. The data described in Figure 5 were analyzed to obtain combination drug indices (CDI), using the CalcuSyn software to determine whether the effects of the combination treatments demonstrated a synergistic effect (CDI < 1), an additive effect (CDI = 1), or an antagonistic effect (CDI > 1).

Combination of AE-CA or CPT	HEC-1A		HEC-1B		
	AE-CA	CPT	AE-CA	CPT
Cisplatin(*μ*M)				
1	0.201	0.163	0.605	0.635
5	0.116	0.161	0.173	0.165
10	0.177	0.256	0.254	0.237
20	0.284	0.388	0.522	0.464
30	0.382	0.488	0.814	0.761
40	0.464	0.533	1.081	1.021
50	0.563	0.623	1.375	1.316
